# The polyphyly of *Plasmodium*: comprehensive phylogenetic analyses of the malaria parasites (order Haemosporida) reveal widespread taxonomic conflict

**DOI:** 10.1098/rsos.171780

**Published:** 2018-05-23

**Authors:** Spencer C. Galen, Janus Borner, Ellen S. Martinsen, Juliane Schaer, Christopher C. Austin, Christopher J. West, Susan L. Perkins

**Affiliations:** 1 Sackler Institute for Comparative Genomics, American Museum of Natural History, Central Park West at 79th St., New York, NY 10024, USA; 2 Richard Gilder Graduate School, American Museum of Natural History, Central Park West at 79th St., New York, NY 10024, USA; 3 Institute of Zoology, Biocenter Grindel, University of Hamburg, Martin-Luther-King-Platz 3, D-20146 Hamburg, Germany; 4 Center for Conservation Genomics, Smithsonian Conservation Biology Institute, National Zoological Park, PO Box 37012, MRC5503, Washington, DC 20013-7012, USA; 5 Department of Biology, Humboldt University, 10115, Berlin, Germany; 6 Department of Biological Sciences, Museum of Natural Science, Louisiana State University, Baton Rouge, LA 70803, USA; 7 Wildlife Program, Yurok Tribe, Klamath, CA 95548, USA

**Keywords:** *Plasmodium*, malaria, phylogeny, base composition bias, polyphyly

## Abstract

The evolutionary relationships among the apicomplexan blood pathogens known as the malaria parasites (order Haemosporida), some of which infect nearly 200 million humans each year, has remained a vexing phylogenetic problem due to limitations in taxon sampling, character sampling and the extreme nucleotide base composition biases that are characteristic of this clade. Previous phylogenetic work on the malaria parasites has often lacked sufficient representation of the broad taxonomic diversity within the Haemosporida or the multi-locus sequence data needed to resolve deep evolutionary relationships, rendering our understanding of haemosporidian life-history evolution and the origin of the human malaria parasites incomplete. Here we present the most comprehensive phylogenetic analysis of the malaria parasites conducted to date, using samples from a broad diversity of vertebrate hosts that includes numerous enigmatic and poorly known haemosporidian lineages in addition to genome-wide multi-locus sequence data. We find that if base composition differences were corrected for during phylogenetic analysis, we recovered a well-supported topology indicating that the evolutionary history of the malaria parasites was characterized by a complex series of transitions in life-history strategies and host usage. Notably we find that *Plasmodium*, the malaria parasite genus that includes the species of human medical concern, is polyphyletic with the life-history traits characteristic of this genus having evolved in a dynamic manner across the phylogeny. We find support for multiple instances of gain and loss of asexual proliferation in host blood cells and production of haemozoin pigment, two traits that have been used for taxonomic classification as well as considered to be important factors for parasite virulence and used as drug targets. Lastly, our analysis illustrates the need for a widespread reassessment of malaria parasite taxonomy.

## 1. Introduction

A complete understanding of human pathogenic diseases is dependent on robust phylogenetic hypotheses to determine the evolutionary origins of the parasitic organisms that cause them [1–4]. Informative phylogenies are dependent on thorough taxon sampling for not only accurate phylogenetic inference [5–7], but also accurate reconstruction of trait evolution [8], and in the case of parasites, dense taxon sampling is critical for reconstructing histories of transitions among host groups that led to the origin of disease [9–11]. Complicating this matter is the observation that many parasite groups are often rare, difficult to sample and may be challenging to generate large sequence datasets for [12], resulting in an ambiguous understanding of the origins of many parasitic diseases.

The malaria parasites (order Haemosporida) are well known for being a devastating scourge of human health with five species known to infect humans, though broadly the order consists of over 500 described species from at least 15 genera that infect mammals, birds, chelonians and squamates throughout the world and are transmitted by several clades of blood-feeding dipteran insects [13,14]. Both the taxonomic classifications and hypotheses of evolutionary relationships among the major lineages within the Haemosporida have been controversial and fluid along the entire history of work on this group [13]. Once molecular data from the parasites became more readily available for phylogenetic analyses, widely different scenarios have been presented, all highly inconsistent and dependent on taxon sampling, type of molecular characters included, and the analytical approach used [14]. As a result, the evolution of key life-history characters and transitions among host groups remain poorly understood. For instance, recent molecular phylogenies have suggested opposing histories for such traits as asexual reproduction in the host bloodstream, a character that is thought to contribute to malaria virulence [15], as well as the number of switches that have occurred among sauropsid and mammalian hosts [16–18].

A long-standing problem for broad systematic analyses of malaria parasites has been the difficulty of achieving a balance between obtaining representative taxonomic coverage from across this diverse and often difficult to sample order and generating enough characters for robust phylogenetic estimation [14]. Early molecular phylogenies generated gene trees and focused almost exclusively on the genus of human medical concern, *Plasmodium* [19–23]. Later studies incorporated samples from multiple genera within the Haemosporida [16,24,25], eventually expanding taxon sampling to include poorly studied and enigmatic haemosporidian genera such as *Haemocystidium*, *Hepatocystis*, *Polychromophilus* and *Nycteria* [18,26–30]. Although these studies benefited from expanded taxon sampling across the order, character sampling still largely remained limited to organellar (mitochondrial and apicoplast) sequence data with each of the aforementioned analyses sequencing no more than a single nuclear locus. Furthermore, the topologies produced by these studies vary widely and thus appear to be highly sensitive to using different taxon sets and analytical approaches.

The instability of the phylogenetic hypotheses for the Haemosporida has resulted in various conclusions concerning the rooting of the haemosporidian tree. When rooted with other apicomplexan taxa, such as *Babesia* or *Theileria*, the earliest diverging haemosporidian lineage has usually been the avian-infecting genus *Leucocytozoon* [24,31]. However, other analyses that implemented outgroup-free methods instead recovered topologies showing haemosporidians divided into two major clades, one containing avian and saurian-infecting parasites and some bat-infecting genera, and the other containing all other mammalian parasites, including all species that infect humans [17,29]. The large variation observed across haemosporidian topologies has also resulted in uncertainty regarding the monophyly of several genera, particularly the genus *Plasmodium*. *Plasmodium* has been found to be paraphyletic [16] or polyphyletic [18,28] across different studies, demonstrating a clear need for a comprehensive analysis that combines broad taxon and character sampling.

The question of how many times haemosporidians may have switched from birds to mammals has also been contentious. The earliest broad phylogeny suggested that there was just one switch each into sauropsids and mammals, with all sauropsid and mammalian parasites forming divergent clades [24]. However, recent studies that incorporated much more rigorous sampling of malaria parasites from bats and ungulates have complicated estimates of the number of host-switching events. Several studies have recovered parasites from bats as closely related to avian-infecting *Plasmodium* species, implying that there were at least two transitions to mammals [32,33]. Yet subsequent analyses that used broad sampling of bat haemosporidians have failed to recover this pattern, finding further evidence for single switches to sauropsid and mammalian hosts [18,28]. In three recent studies that focused on ungulate malaria parasites, the authors recovered inconsistent patterns of host-switching among studies despite similar taxon and gene sampling [29,30,34].

Despite the clear need for genome-wide data, generating large sequence datasets for non-model haemosporidian species has proven challenging [35,36], and as a result phylogenomic analyses of the malaria parasites have largely remained taxonomically restricted to the genus *Plasmodium* ([37–39], though see [31] and [40]). Large, multi-locus studies of the malaria parasites are also hindered by the problem of conflicting phylogenetic signal produced by heterogeneous base composition biases [41,42]. Malaria parasite genomes are characterized by extreme but heterogeneous base compositions. For instance, the genome of the virulent human malaria parasite *Plasmodium falciparum* has a GC content of approximately 19% [43], though GC content can be as high as 42% in another human-infecting species, *Plasmodium vivax* [44,45]. Base composition bias has the potential to mislead phylogenetic inference when composition has converged in evolutionarily distant clades [46–49], which can be compounded when the outgroup differs in compositional bias from all or part of the ingroup [50,51]. Base composition bias is also strongly associated with codon usage and amino acid composition [52–54], which can potentially mislead phylogenetic inference due to convergence within protein-coding genes [55–57].

Systematists have long recognized that the difficulties of estimating the phylogeny of the Haemosporida will only be ameliorated by analyses that incorporate broad taxon sampling and genome-wide multi-locus datasets [14,35]. Here, we improve upon previous analyses by incorporating numerous taxa that have been poorly represented in previous analyses or have never been analysed within a phylogenetic framework before, including four different genera of parasites that infect bats, parasites from ungulates and an array of malaria parasite taxa from sauropsid hosts. We also included all species that infect humans, using data from the recent releases of both the *Plasmodium malariae* and the *Plasmodium ovale* genomes [58]. We interrogate genome-wide multi-locus data using a broad array of data subsets and analytical (supermatrix concatenation and species tree) approaches, testing the extent to which convergence in base composition can mislead phylogeny. In total, we present here the most comprehensive phylogeny of the malaria parasites estimated to date.

## 2. Material and methods

### 2.1. Sample acquisition, DNA sequencing and sequence alignment

We generated novel sequence data for 34 haemosporidian taxa (electronic supplementary material, table S1). Samples in the form of dried blood spots, buffered blood or tissues were collected by the authors or donated. We extracted DNA using either the DNeasy Blood and Tissue Kit (Qiagen) or the Mag-Attract High-Molecular-Weight DNA Kit (Qiagen) according to the manufacturer's protocols. For each sample that we determined to be positive for haemosporidian infection by microscopic examination, we attempted to amplify 21 nuclear protein-coding genes (electronic supplementary material, table S2). We chose to sequence exclusively nuclear loci due to the unusual characteristics of haemosporidian mitochondrial genomes that may influence their utility for phylogenetic analysis, namely the linearly concatenated structure that has been hypothesized to result in concerted evolution of this genome [59,60] and the recent finding of large rearrangements and gene losses in some lineages [61]. Using the primers previously described in [31] with 1 µl template DNA, 10 µl TopTaq Master Mix (Qiagen), 6 µl H_2_O and 1 µl each primer (10 mM). Each gene was amplified using a nested approach, with both outer and inner reactions consisting of an initial 30 s at 94°C, followed by 35 cycles of 94°C for 30 s, 47°C for 30 s and 62°C for 45 s, with a final 90 s extension at 62°C. We used 1 µl of PCR product from the outer reaction as template for the inner reaction. All inner primers incorporated CAG (5′-CAGTCGGGCGTCATCA-3′) or M13R (5′-GGAAACAGCTATGACCAT-3′) tags, which were used for Big Dye (Life Technologies, Foster City, CA) cycle sequencing on an ABI 3730 DNA Analyzer. We generated contigs and edited sequences in Geneious v. 8.05.

We combined these sequence data with previously published sequences for eight haemosporidian taxa from [31], as well as 16 additional species in the genus *Plasmodium* from the PlasmoDB database [62]. Orthologues were identified by reciprocal BLAST hits with an e-value cut-off of 10^−10^. Based on the finding of [31] that haemosporidian phylogeny is robust to the choice of apicomplexan outgroup, we used *Theileria annulata* from Piroplasmida, the sister group to the Haemosporida, as the outgroup [63]. In sum, this dataset includes 58 ingroup taxa from 7 currently named haemosporidian genera. We aligned nucleotide and translated amino acid sequences for each gene individually using the L-INS-i algorithm implemented in MAFFT [64]. We modified the MAFFT nucleotide alignments for further analysis using TranslatorX [65] to generate codon alignments guided by the amino acid translation that maintains the reading frame for all loci.

### 2.2. Base composition and codon usage bias

We calculated base composition summary statistics in R [66] using the package *seqinR* [67]. To characterize codon usage bias across the haemosporidian phylogeny, we calculated relative synonymous codon usage (RSCU) using *seqinR* and the effective number of codons (ENCs) used with the program CodonW [68] for each taxon. RSCU calculates how over- or under-used an individual codon is relative to random expectations, and varies from zero (codon not used) to six (one codon is used exclusively for a sixfold degenerate amino acid; [69]). Similarly, ENC measures departures from equal usage across all synonymous codons, and varies from 20 (only one codon is used for each amino acid) to 61 (alternative synonymous codons are equally likely; [70]). We plotted variation in codon usage using principal components analysis implemented in R, and visualized the evolution of base composition across phylogenies using the maximum-likelihood ancestral state estimator contMap function in the *phytools* package [71]. We tested the fit of the evolution of base composition to an Ornstein–Uhlenbeck (OU) process, and evaluated whether there is evidence for shifts in the OU process across the phylogeny using the OUshifts function in the *phylolm* package [72]. Compositional heterogeneity in nucleotide alignments was measured using the relative composition frequency variability (RCFV) statistic [73], which measures the deviation from the mean for amino acids and nucleotides and sums these values across taxa, with higher values indicating increased compositional heterogeneity in a given partition. We used phylogenetic ANOVA implemented in the R package *geiger* [74] to test for differences in GC content among clades.

### 2.3. Phylogenetic methods and analytical approach

We analysed both codon and amino acid alignments using a Bayesian approach implemented in BEAST v. 1.8 [75] and ML implemented in RAxML v. 8 [76]. We analysed these datasets using fully concatenated supermatrices (i.e. all genes and sites evolving according to the same model) and partitioned supermatrices to determine whether partitioning altered the effects of base composition bias. All analyses were repeated using codon alignments and amino acid alignments in both Bayesian and ML frameworks. We tested for a global molecular clock using likelihood ratio tests for each individual gene. Best fit models of evolution were estimated using PartitionFinder v. 2.1.1 ([77]; electronic supplementary material, table S3). RAxML analyses were run using 100 bootstrap replicates. BEAST analyses were run through the CIPRES portal [78] using a Yule tree prior, and analyses were repeated using both strict and lognormal relaxed molecular clocks (see electronic supplementary material, table S4 for all phylogenetic analyses performed). For all Bayesian analyses we used Tracer v. 1.6 [79] to assess convergence, determine that all ESS values were greater than 200, and identify the appropriate burn-in. To test the effect of missing data, we ran a partitioned analysis in BEAST using amino acid data and a relaxed molecular clock but with a reduced taxon set of 21 species that included only the most complete (number of sequenced loci) samples for each lineage.

We also analysed codon and amino acid datasets using a species tree approach. Species tree methods typically assume that gene tree discordance is due to incomplete lineage sorting, though it is possible for systematic error to occur in the estimation of gene trees due to base composition bias. To test the impact of base composition on species tree inference, we estimated phylogenies using a species tree approach implemented in ASTRAL-II [80]. ASTRAL-II contrasts with supermatrix methods by accounting for incomplete lineage sorting under the multispecies coalescent model using unrooted gene trees and was chosen for its ability to handle missing data and its accuracy in simulations [80,81]. For this analysis, we estimated individual gene trees for codon and amino acid alignments for all ASTRAL-II analyses using the same parameters described above in RAxML v. 8.

## 3. Results

### 3.1. Base composition and codon usage bias

**Figure 1.**
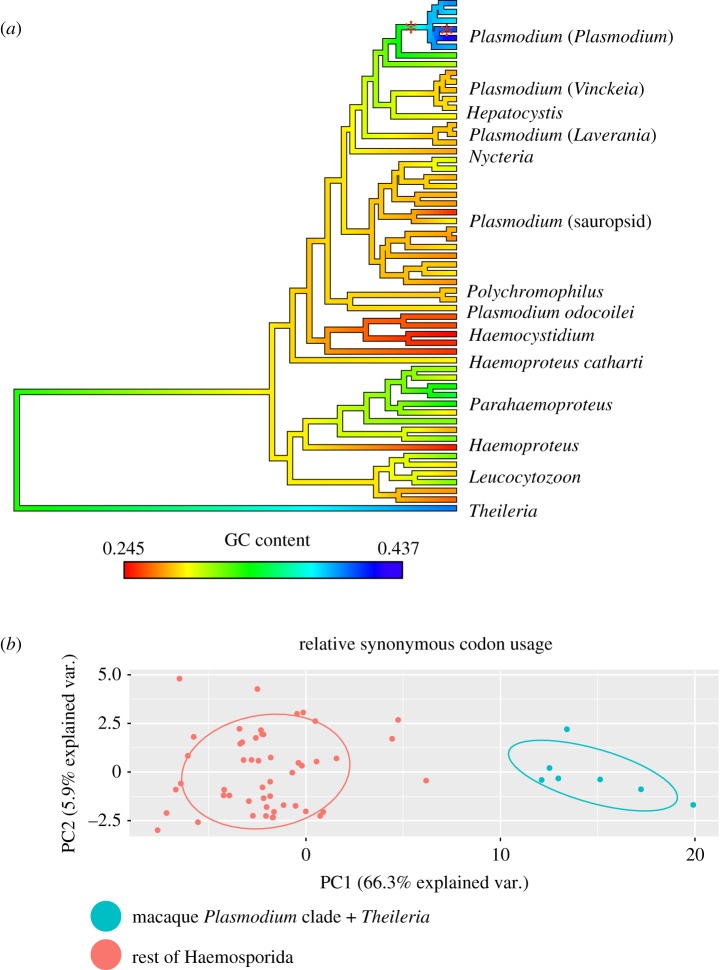
The order Haemosporida is characterized by extremely heterogeneous base composition and codon usage. (*a*) The evolution of GC content is shown optimized across the haemosporidian phylogeny using the contMap function in *phytools*. Note that the macaque *Plasmodium* clade has evolved a GC content that is significantly higher than the rest of the ingroup. Branches where significant shifts in GC content occurred as determined by the OUshifts analysis are denoted by asterisks. (*b*) Principle components analysis of RSCU across the Haemosporida. The macaque *Plasmodium* clade groups with the outgroup *Theileria* to the exclusion of the other malaria parasites, indicating similar usage of synonymous codons within degenerate amino acids.

The complete phylogenetic dataset consisted of 21 loci for 58 ingroup taxa and one outgroup, with codon and amino acid alignments consisting of 19 851 and 6747 aligned sites, respectively (table 1). Dataset completeness was uneven across samples and genera, varying from an average of 8 loci per sample in *Polychromophilus* (range: 5–11) to 17.5 loci per sample in *Plasmodium* (range: 6–21). However, with the exception of *Polychromophilus* and *Nycteria* all genera were represented by at least one sample with sequence data for 15 genes. All sequences generated by this study have been deposited in GenBank (see electronic supplementary material, table S2 for accession numbers). Base composition was highly variable among taxa in our dataset (figure 1; electronic supplementary material, figure S1), with mean GC content across loci ranging from 24.5% (*Plasmodium mackerrasae*) to 43.7% (*P. vivax*). Variation in GC content of third codon positions (GC3) was even more extreme, varying from just 4.5% (*Haemocystidium* (*Simondia*) *metchnikovi*) to 49.5% (*P. vivax*). Just six ingroup species, all members of a clade of *Plasmodium* that infect primarily macaques (*P. vivax, P. inui, P. cynomolgi, P. knowlesi, P. coatneyi* and *P. fragile*; hereafter ‘macaque *Plasmodium*'), had GC3 content greater than 27%. The mean GC3 content for the macaque *Plasmodium* clade was 41.2%, similar to the GC3 content of the outgroup *Theileria* (39.9%). Mean GC and GC3 were significantly higher in the macaque *Plasmodium* clade than the rest of the Haemosporida (phylogenetic ANOVA: GC content *F*_1, 56_ = 160.9, *p* < 0.001; GC3 content *F*_1, 56_ = 179.64, *p* < 0.001). The divergent base composition profile in the macaque *Plasmodium* clade was supported by the OUshifts analysis that identified two shifts in GC content across the phylogeny, one on the branch at the base of the macaque *Plasmodium* clade and another on the terminal branch of *P. vivax* (figure 1). The RCFV value for the entire dataset (0.071) was more than an order of magnitude greater than for any individual taxon (0.005), indicating that base composition heterogeneity occurred among taxa rather than among genes within taxa.

**Table 1.** Summary data for nucleotide and amino acid datasets used in this study.

dataset	taxa	aligned length	% gaps	% missing data
codon alignment	59	19 851 bp	12.2	31.7
codon Position (1 + 2)	59	13 234 bp	12.2	31.7
amino acid	59	6747 aa	14.4	30.5

Consistent with trends observed in base composition, we found codon usage to be heavily biased towards AT-rich codons across all haemosporidians with the exception of the macaque *Plasmodium* clade and the outgroup *Theileria* (figure 1). The average ENCs used (with 20 indicating extremely biased codon usage and 61 indicating even codon usage) was 49.9 within the macaque *Plasmodium* clade, in contrast to 33.6 for the rest of the malaria parasites. The average ENC for *Theileria* was 48.0, and multivariate analyses showed that the RSCU profile of the macaque *Plasmodium* was more similar to that of the outgroup *Theileria* than to other haemosporidians (figure 1). ENC was significantly higher in the macaque *Plasmodium* clade than the rest of the Haemosporida (phylogenetic ANOVA: *F*_1,56_ = 140.3, *p* < 0.001).

### 3.2. Topological consistencies across phylogenetic analyses

**Figure 2.**
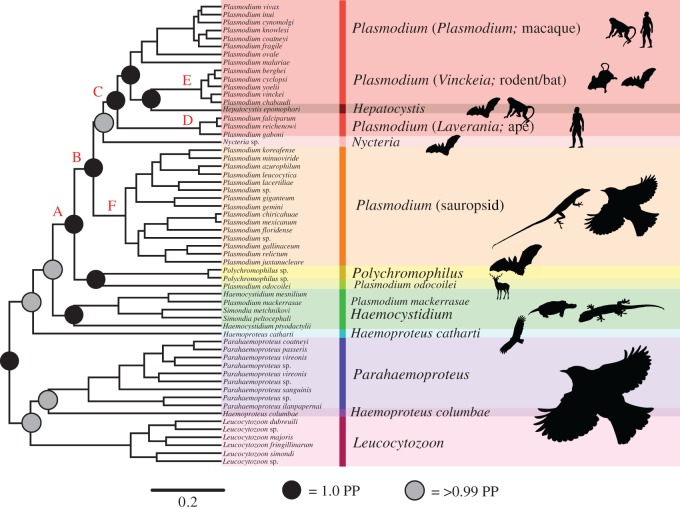
The favoured haemosporidian phylogeny. The haemosporidian parasite phylogeny recovered from BEAST using the fully partitioned amino acid dataset and lognormal relaxed molecular clock. The outgroup *Theileria* is not shown for ease of viewing the ingroup topology. Shown as silhouettes are representatives of the vertebrate host group for each haemosporidian lineage. Clades denoted with the letters A–F are referred to in the Discussion.

We found strongest and most consistent support (12 different analyses) for a clade consisting of *Leucocytozoon*, *Haemoproteus* and *Parahaemoproteus* as the root taxon sister to the rest of the Haemosporida, though four different analyses recovered only *Leucocytozoon* at this position (figure 2; electronic supplementary material, table S4). The genus *Haemocystidium*, which has at times been hypothesized to be a subgenus of *Haemoproteus* [26], was found in all analyses to be distinct from avian *Haemoproteus* and *Parahaemoproteus*, forming a divergent clade at the base of the mammalian haemosporidian radiation. In nearly all analyses *Haemocystidium* was either closely allied with or sister to a parasite isolated from a turkey vulture, identified as *H. catharti* [82]. *Haemoproteus catharti* was consistently recovered outside of the primary *Haemoproteus* + *Parahaemoproteus* clade, rendering *Haemoproteus* polyphyletic (figure 2; electronic supplementary material, table S4). We tested whether *H. catharti* is a member of the same lineage as *Haemoproteus antigonis*, a divergent avian malaria parasite recently isolated from cranes that has been hypothesized to represent a novel haemosporidian genus [83]. Using cytochrome *b* data for a subset of avian malaria parasites included in this study in addition to the cytochrome *b* data from Bertram *et al*. [83] (GenBank accession numbers: KX223839-KX223844), we estimated a gene tree using RAxML with the aforementioned parameters and partitioning by codon position. We found that *H. catharti* and *H. antigonis* are not monophyletic, with all *H. antigonis* samples forming a clade to the exclusion of all other haemosporidian genera and *H. catharti* grouping with *Haemocystidium* (electronic supplementary material, figure S2).

We found that the genus *Plasmodium* was polyphyletic in all analyses (electronic supplementary material, table S4). Species or clades previously classified as *Plasmodium* were found to be more closely related to other haemosporidian genera in four instances: (1) *Plasmodium mackerrasae* was nested within *Haemocystidium* in all analyses; (2) *Plasmodium odocoilei*, the parasite from North American white-tailed deer, was recovered outside of the primary mammal *Plasmodium* clade in all analyses as sister to the bat-infecting genus *Polychromophilus*; (3) the rodent and bat *Plasmodium* clade was sister to *Hepatocystis* in all analyses; and (4) *Nycteria* was recovered as either sister to the primary mammal *Plasmodium* clade (containing ape, rodent and bat *Plasmodium* in addition to *Hepatocystis*) or sister to sauropsid *Plasmodium* (electronic supplementary material, table S4). We found support for *Nycteria* as sister to the mammalian *Plasmodium* + *Hepatocystis* clade almost exclusively in amino acid analyses; tests of a global molecular clock were rejected for all loci, and we found that using a lognormal relaxed molecular clock favoured the recovery of *Nycteria* as sister to the mammal *Plasmodium* clade (electronic supplementary material, table S4).

As two recent studies based on mitochondrial sequence data have also recovered haemosporidian parasites of ungulates as more closely related to *Polychromophilus* than to other mammal-infecting *Plasmodium* [29,34], we sought to test whether the *P. odocoilei* isolate included in this study is a member of the same clade as the previously studied ungulate malaria parasites. To do so we used the cytochrome *b* sequence data of malaria parasites isolated from ungulates from [29] and [34] (GenBank accession numbers: LC090214, LC090215, KT367830, KT367841 and KT367842) in addition to cytochrome *b* data from *P. odocoilei* and all major haemosporidian lineages included in this study to estimate a cytochrome *b* gene tree using RAxML with the aforementioned input parameters and partitioning by codon position. This analysis recovered *P. odocoilei* nested within a clade containing all other haemosporidians previously recovered from ungulates, indicating that the *Plasmodium* parasites that infect ungulates represent an unnamed divergent haemosporidian lineage (electronic supplementary material, figure S3).

### 3.3. Testing the effect of base composition bias on supermatrix and species tree analyses

**Figure 3.**
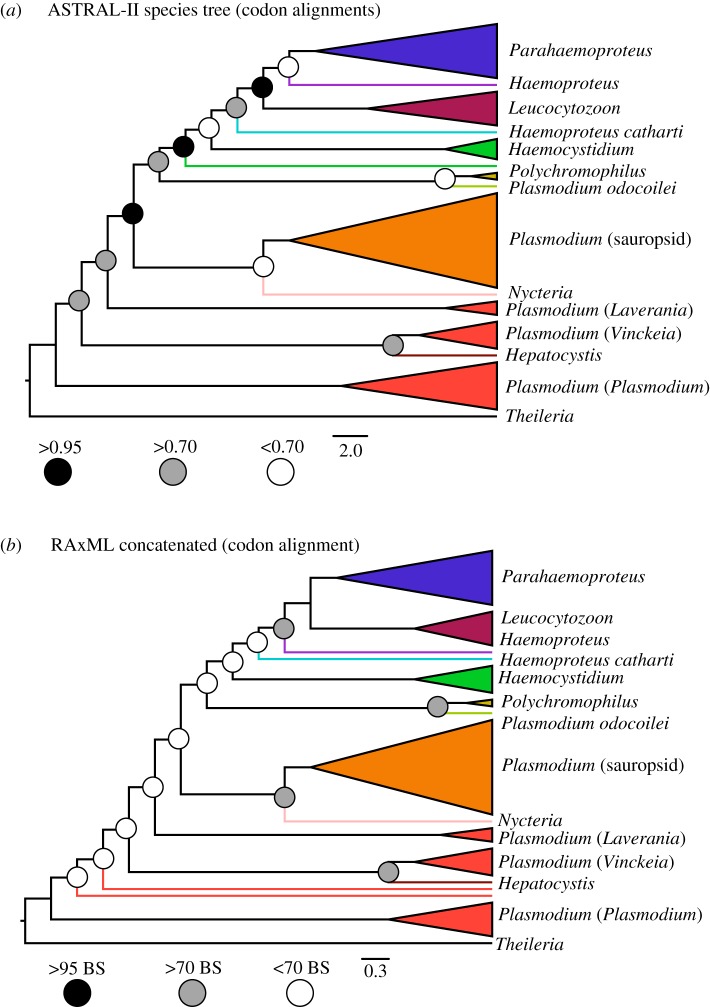
The ‘mammal-first' topology is likely driven by base composition convergence between the macaque *Plasmodium* clade and the outgroup *Theileria*. Two analyses recovered the ‘mammal-first' topology: (*a*) the ASTRAL-II species tree analysis using gene trees generated from nucleotide codon alignments, and (*b*) RAxML supermatrix analysis of the unpartitioned codon alignment. This topology contrasts dramatically with the topologies recovered from analyses that correct for the base composition convergence effect, suggesting the ‘mammal-first' topology is an artefact.

We observed that two analyses, the unpartitioned analysis using the codon alignment and the ASTRAL-II species tree analysis using gene trees generated from codon alignments, produced topologies that differed dramatically from the topologies we recovered in all other analyses (figure 3). The aforementioned analyses using codon alignments generated similar topologies that we will refer to as the ‘mammal-first' topology, which recovered the macaque *Plasmodium* clade at the root of the phylogeny as sister to the rest of the Haemosporida. As the ‘mammal-first' topology deviated significantly from all other analyses, which universally recovered the avian malaria parasite genera *Leucocytozoon*, *Haemoproteus* and *Parahaemoproteus* at the root of the tree, we sought to test the robustness of the ‘mammal-first' phylogenetic hypothesis and determine whether it may be an artefact of non-phylogenetic signal caused by base composition heterogeneity. We hypothesized that the ‘mammal-first' topology may have been driven by base composition convergence between the macaque *Plasmodium* clade and the outgroup *Theileria*, resulting in ‘GC attraction' driving this clade of *Plasmodium* towards the root. We tested this hypothesis by running three additional phylogenetic analyses that sought to correct for the base composition attraction effect: (1) an unpartitioned analysis using the codon alignment with the macaque *Plasmodium* clade removed, (2) an unpartitioned analysis using the full taxon set but with the highly GC-heterogeneous third codon positions removed, and (3) an outgroup-free (*T. annulata* removed) relaxed molecular clock analysis in BEAST following [17], who recovered a topology similar to the ‘mammal-first' tree using an outgroup-free approach. We found that none of the additional analyses recovered the ‘mammal-first' topology; rather, in each case the analyses recovered topologies with the mammalian malaria parasites derived from sauropsid-infecting ancestors (electronic supplementary material, table S4). The observation that the ‘mammal-first' topology was generally poorly supported and is only recovered in analyses that provide no correction for base composition bias and include both the macaque *Plasmodium* clade and the outgroup strongly suggests that this topology is an artefact due to base composition convergence.

As we also observed the divergent ‘mammal-first' topology in ASTRAL-II species tree analyses using gene trees generated from codon alignments (electronic supplementary material, figure S4), we sought to test the artefactual nature of this topology in a similar manner to that described above for supermatrix analyses. To test the hypothesis that error in gene tree estimation due to base composition bias was responsible for the ‘mammal-first' topology in species tree analyses, we generated gene trees using amino acid datasets. We found that support for the ‘mammal-first' topology disappeared when using amino acid derived gene trees; while codon alignments resulted in 10 gene trees that produced the macaque *Plasmodium* clade as sister to the rest of the Haemosporida, amino acid alignments resulted in just one gene tree with this topology. The species trees generated using amino acid data recovered a large sauropsid haemosporidian clade at the root that included *Haemocystidium* in addition to *Leucocytozoon*, *Haemoproteus* and *Parahaemoproteus*, though this tree was weakly supported (electronic supplementary material, figure S4).

## 4. Discussion

### 4.1. The phylogeny of the order Haemosporida

Our comprehensive phylogenetic analyses recovered strong support for a novel view of malaria parasite evolution that provides a framework for understanding haemosporidian life-history traits, host-switching, diversification and the origin of the malaria parasites that infect humans. These analyses resolve several historically controversial relationships among the major genus-level lineages within the Haemosporida, though they also challenge many of the current taxonomic classifications.

The genus *Plasmodium* has consistently been found to be non-monophyletic in all molecular phylogenies with broad haemosporidian taxon sampling [16–18,24,28,30], with various analyses recovering the genus as either paraphyletic or polyphyletic. The present analyses confirm the polyphyly of *Plasmodium* with high support on all relevant nodes as species currently classified as *Plasmodium* were found to be more closely related to other haemosporidian genera in four instances. The first instance of polyphyly involves the position of the Australian lizard malaria parasite, *Plasmodium mackerrasae*, within the clade of *Haemocystidium* parasites. In two previous analyses using the same sample [16,24] this species also did not group with other lizard *Plasmodium* species; however, those analyses did not include any members of *Haemocystidium*. The species as originally described [84] clearly showed schizonts in blood cells, whereas *Haemocystidium* species do not. Though it remains possible that this sample contained a mixed infection with *Haemocystidium*, we observed no signs of a mixed infection such as ‘multiple peaks' in sequence chromatograms and this sample grouped with *Haemocystidium* in all gene trees for which we had sequence. Although this finding suggests that *P. mackerrasae* is incorrectly classified, we were unable to study the morphology of this sample and so we do not recommend any taxonomic changes for this species until new and verified material can be obtained.

With the exception of *P. odocoilei* (which we discuss below), the status of the rest of the taxa currently ascribed to the genus *Plasmodium* presents a vexing taxonomic problem and we are forced to confront several possible solutions, none of which is particularly straightforward. A major complication is the finding that the genera *Hepatocystis* and *Nycteria* are nested within a large clade of species that are currently classified as *Plasmodium* that infect lizards and birds as well as primates, rodents and bats (Clade B, figure 2). The position of *Hepatocystis* nested within the mammalian *Plasmodium* (Clade C, figure 2) presents a particularly challenging problem, as this genus lacks several diagnostic traits that are characteristic of *Plasmodium* such as erythrocytic schizogony [85,86] and *Hepatocystis* is vectored by ceratopogonid midges of the genus *Culicoides* rather than mosquitoes [87]. Given that our analyses are not the first to find this grouping of *Hepatocystis* with the mammalian *Plasmodium* species [23,24,88], and with the expanded data analysed here, this relationship seems increasingly solid. One possible solution to deal with the position of *Hepatocystis* would be to revise the taxonomy of this clade substantially to enforce that both *Hepatocystis* and *Plasmodium* are monophyletic. As *P. malariae* is the type species of the genus *Plasmodium*, this change would require that the clade of ape malaria parasites (Clade D, figure 2) that includes *P. falciparum* be changed to *Laverania* (re-elevated from subgenus to genus; [89]) and the clade of rodent malaria parasites (Clade E, figure 2), which are widely used as model systems would likewise need to be changed to *Vinckeia*, their current subgeneric classifier. Both of these changes would undoubtedly be extremely unpopular and thus probably ignored. A second alternative is to lump the genus *Hepatocystis* within *Plasmodium*. This solution would prevent complicated name changes, which is advantageous; however, it would also mean that the defining characters of the genus *Plasmodium* would need to be revised to the point where they are so broad that they are not useful in delineating the genus. For instance, if *Hepatocystis* parasites were to be reclassified as *Plasmodium* the resulting genus would include parasites that are vectored by either ceratopogonid midges or mosquitoes, and would remove erythrocytic schizogony as a defining trait of the group.

A similar conflict is produced by the position of *Nycteria* as the sister group to the mammalian *Plasmodium* + *Hepatocystis* clade to the exclusion of the clade of sauropsid-infecting *Plasmodium* (Clade F, figure 2). The genus *Nycteria* was originally erected because these parasites of bats did not consistently show characteristics of *Plasmodium*, *Hepatocystis* or *Polychromophilus*, with only gametocytes present in the blood and with liver stages similar to those seen in *P. falciparum* [90]. The taxonomy of *Nycteria* poses a similar problem to that posed by *Hepatocystis*: lumping *Nycteria* within *Plasmodium* would eliminate the diagnostic traits of *Plasmodium*, while the retention of *Nycteria* at the genus level would require the elevation of a new genus for the sauropsid *Plasmodium* clade. There is, in fact, a different generic name with precedence for the saurian and avian parasites—*Haemamoeba*—so the most straightforward path to take would be to resurrect this genus and use it for the lizard and bird parasites currently called *Plasmodium*. We suspect that this would also be unpopular and largely ignored by those working on these groups, however, which will ultimately create more confusion in the field.

A potentially unifying solution to the taxonomic conflict observed here is to encourage the use of the subgeneric names for these clades as much as possible, to reflect that they are distinct lineages and to take into consideration the evolutionary relationships. The issues of changing the name of *Plasmodium*, particularly with respect to *P. falciparum*, was remarked upon by Garnham [13] who noted that it was seen as ‘unwelcome'—even when the ICZN had sanctioned via a special ruling for *Laverania* in its place. As a consolation, though, the historic use of subgenera within the Haemosporida has been common to reflect the distinctiveness of certain taxa and groups [13,14]. Interestingly, we note that the taxonomic challenge posed by the Haemosporida strikingly parallels that faced by entomologists working out the systematics of *Drosophila*. The type species of the genus, *Drosophila funebris*, belongs to a different clade than the very well-known model organism, *Drosophila melanogaster*, which alternatively has been classified in the genus *Sophophora* [91,92]. Rather than dramatically alter the taxonomy of a model organism, these authors favoured using evolutionary relationships as opposed to strict taxonomy for referring to species and groups. O'Grady & Markow [92] favoured this scenario as it would cause the least amount of disruption and confusion, with all commonly studied species retaining their current generic name and leaving open the ability to make changes in the future when new data become available. Thus, we argue here that the ‘evolutionary taxonomic’ approach to dealing with the polyphyly of *Plasmodium* is probably the best solution to the present conflict. We recognize fully that with the vast number of researchers working on *P. falciparum*, the rodent malaria model species, as well as the globally widely studied avian malaria parasites—not to mention the voluminous literature that already exists that uses the genus *Plasmodium* for these species—it is highly impractical to change their generic designation at this point. However, it is also important to acknowledge the many differences in life histories, morphology and vector usage by *Hepatocystis*, *Nycteria, Polychromophilus* and several of the sauropsid haemosporidian parasites that use phlebotomine sandflies as vectors (*P. mexicanum*), or lack haemozoin pigment (e.g. *P. azurophilum*, *P. leucocytica*).

The final instance of polyphyly that we observed in *Plasmodium* dealt with *P. odocoilei* and other ungulate malaria parasites, which we recovered as sister to *Polychromophilus*. One possible taxonomic solution is to further extend the generic boundary of *Plasmodium* to include all of what is Clade A (figure 2), so as not to alter the taxonomic name of this lineage and to treat *Polychromophilus* as an evolutionarily distinct lineage within *Plasmodium* similar to *Hepatocystis* and *Nycteria*. The other is to give a new generic name for the ungulate malaria, for which there is not any clear precedent. Our analysis based on cytochrome *b* indicates that all recently sequenced ungulate malaria parasites are monophyletic (electronic supplementary material, figure S3), though we recommend that additional multi-locus data are collected from other species within this clade before formal names (e.g. *Plasmodium bubalis* and *Plasmodium caprae*) are changed.

Our analyses also shed light on other questions of the evolutionary history of the haemosporidians, particularly their origin. The earliest divergence within the malaria parasites has recently generated controversy, with studies supporting either early diversification among malaria parasites that infect birds [16,31] or a deep split between mammal and sauropsid-infecting malaria parasite clades [17]. We found that after correcting for base composition biases there was uniform support for the avian malaria parasites *Leucocytozoon*, *Haemoproteus* and *Parahaemoproteus* as the earliest diverging lineages within the Haemosporida, indicating that avian hosts were probably ancestral. The majority of our analyses (12 of 17 analyses that were not misled by base composition) supported an avian malaria clade containing *Leucocytozoon*, *Haemoproteus* and *Parahaemoproteus* as sister to the rest of the malaria parasites, though in a minority of analyses (4 of 17) we found just *Leucocytozoon* at this position. It is notable that another recent study of haemosporidian phylogeny based on complete mtDNA genomes [93] recovered strong support for *Leucocytozoon* as sister to the rest of Haemosporida. As the pattern of divergence among *Leucocytozoon*, *Haemoproteus* and *Parahaemoproteus* was not consistent across the analyses conducted here and in other recent studies, we anticipate that expanded genomic sampling will be required to definitively resolve this relationship.

This study supports *Haemoproteus*, *Parahaemoproteus* and *Haemocystidium* as distinct and evolutionarily divergent genera. While *Haemoproteus* and *Parahaemoproteus* have been previously considered by some authors to be subgenera within the same genus [15], we consistently found a deep divergence between the two lineages indicative of a long history of evolutionary independence. Furthermore, several of our analyses did not recover *Haemoproteus* and *Parahaemoproteus* as sister lineages. The evolutionary distinctiveness of *Haemoproteus* and *Parahaemoproteus* has now been recovered from multiple studies [16,31] and is supported by the observation that these lineages use different vectors: *Haemoproteus* is transmitted by louse flies in the family Hippoboscidae, while *Parahaemoproteus* is transmitted by biting midges in the family Ceratopogonidae [15]. The genus *Haemocystidium*, a malaria parasite of squamates and chelonians that shares numerous traits with *Haemoproteus* and *Parahaemoproteus* [26], was similarly found to be highly divergent from the avian parasites as the basal lineage to the clade containing all mammal malaria. The distinctiveness of *Haemocystidium* has been found previously using primarily mitochondrial data [26,94], though our study is the first to confirm this with multi-locus nuclear sequences.

Our analyses also support the distinctiveness of a parasite collected from a New World vulture (family Cathartidae) in California. Though initially identified as *H. catharti* [82], both the two-gene analysis by Yabsley *et al*. [95] and our multi-locus sequencing here found these parasites to be a divergent lineage and not part of *Haemoproteus* or *Parahaemoproteus*. Similarly, a recent study recovered *Haemoproteus antigonis*, a parasite of cranes (Gruidae) in North America, as a divergent genus-level lineage distantly related to the *Haemoproteus* and *Parahaemoproteus* parasites of other birds [83]. However, we found that based on cytochrome *b* sequences *H. antigonis* is not closely related to the turkey vulture parasite studied here. Taken together, these findings suggest that although several clades of avian malaria parasites may appear similar to *Haemoproteus*, there are deeply divergent yet cryptic lineages of malaria parasites that may be more common than previously appreciated.

Finally, it should be pointed out that these analyses also call into question the status of the family-level classifications in this order. Fallis & Bennett [96], Garnham [13] and Valkiūnas [15] have all advocated for the separation of these parasites into Plasmodiidae, Haemoproteiidae and Leucocytozoodiae based primarily on the specific combination of presence or absence of two primary characters—erythrocytic schizogony and haemozoin pigment. Our most commonly recovered topology could be roughly divided into three distinct families, but the family Plasmodiidae would be inconsistent with the simple scheme first envisioned as some species have sexual stages in dipterans other than mosquitoes, some do not typically show any erythrocytic schizogony, and some taxa do not have pigment. Garnham [13] advocated for using subgeneric names to reflect the observed differences in these groups of haemosporidians; now five decades later and after collecting copious DNA sequence data, we find ourselves with this same basic conclusion.

### 4.2. Host-switching and the evolution of life-history traits

**Figure 4.**
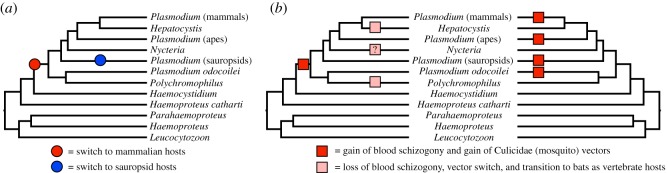
The phylogenetic hypothesis presented here alters our understanding of malaria parasite host-switching and life-history evolution. (*a*) All topologies that we recovered from across phylogenetic analyses that were corrected for base composition bias suggest a single switch to mammalian hosts from sauropsid-infecting ancestors, followed by one additional switch back to sauropsid hosts in the *Plasmodium* lineage. (*b*) This topology suggests two alternative scenarios for the evolution of blood schizogony (asexual reproduction in the host bloodstream) and vector use. One scenario (left) posits a single gain of blood schizogony and a switch to mosquito (Culicidae) vectors in the lineage leading to all mammalian malaria parasites, followed by loss of blood schizogony and a vector switch in three lineages: *Polychromophilus*, *Hepatocystis* and *Nycteria*. Alternatively (right), the four lineages of *Plasmodium* identified in our analysis could have evolved blood schizogony and switched to mosquito vectors independently.

Our confirmation of an avian origin for the malaria parasites has broad implications for our understanding of host-switching within the Haemosporida and the origin of the mammalian malaria parasites (figure 4). The phylogenetic framework supported here suggests just a single origin of mammalian malaria leading to the evolution of the genera *Polychromophilus*, *Nycteria*, *Hepatocystis* and *Plasmodium* from a sauropsid-infecting ancestor. The transition to infecting mammals was followed by a secondary switch back to sauropsid hosts in the *Plasmodium* parasites that infect birds and squamates. The phylogenetic hypothesis produced here also reaffirms the role of bats (order Chiroptera) as major drivers of malaria diversification [18,28]. Malaria parasites appear to have undergone a complex history of divergence following switches to or from bats; our favoured phylogenetic hypothesis suggests either an initial wide radiation of malaria parasites in bats followed by at least four transitions to other host groups (ungulates, primates, rodents and sauropsids) or at least four separate colonizations of bats as hosts (in *Polychromophilus*, *Nycteria*, *Hepatocystis* and mammal *Plasmodium*). The importance of bats in malaria diversification also points to the importance of Asia and Africa as cradles of malaria diversification, as the bat malarias are most diverse and prevalent in these regions [97].

Our analyses also support the finding of [16] that the diversification of major haemosporidian lineages was precipitated by transitions among vectors within the insect order Diptera (true flies). Our understanding of the history of vector transitions is, however, hindered by a lack of basic natural history knowledge as we still are not aware of the vectors for *Haemocystidium* and *Nycteria* (though the *Haemocystidium* subgenus *Simondia* is known to be vectored by horseflies in the genus Tabanidae, [14]). Our favoured hypothesis (figure 4) suggests the possibility that a single switch to mosquito (Culicidae) vectors occurred in parallel with the switch to mammalian hosts, followed by at least two vector transitions in the genera *Polychromophilus* (to nycterbiid batflies) and *Hepatocystis* (to ceratopogonid biting midges) and possibly a third in *Nycteria* (though the vector for this genus is currently unknown).

Lastly, the topology presented here provides a novel understanding of the evolution of asexual reproduction in host blood (exoerythrocytic schizogony). Blood schizogony is exclusive to the genus *Plasmodium*, and has been considered a primary factor contributing to the virulence of this genus [15]. The finding that *Plasmodium* is polyphyletic suggests a dynamic history of gain and loss of blood schizogony. The most parsimonious reconstruction of this trait suggests a gain of schizogony at the transition to mammalian hosts and three independent losses in the lineages leading to *Polychromophilus*, *Nycteria* and *Hepatocystis*, though the causes for the loss of blood schizogony remain unclear. It is worth noting that the loss of blood schizogony appears to have paralleled transitions in vector hosts as well as the primary or exclusive use of bats as vertebrate hosts in *Polychromophilus*, *Hepatocystis* and *Nycteria*, though the biological significance of this correlation is unknown.

### 4.3. The effects of base composition bias and heterogeneity on phylogenetic accuracy

This study provides empirical support to show that the two currently popular approaches for analysing phylogenomic datasets—supermatrix concatenation and species tree methods—are vulnerable to being misled by base composition convergence in distantly related lineages if this bias is not corrected for during analysis. We show here that an incorrect malaria parasite phylogeny that would dramatically alter our understanding of malaria evolution is estimated from both approaches when using nucleotide alignments that have not been corrected for base composition heterogeneity. Supermatrix approaches have previously been justified based on their ability to detect ‘hidden support' that is not contained in any individual partition [98,99], though we show here that strong support for the wrong topology can be recovered due to the overwhelming effect of non-phylogenetic signal produced by non-stationary nucleotide alignments. Similarly, we show that the assumptions of species tree methods, namely that gene trees are estimated accurately and that gene tree discordance is due to incomplete lineage sorting [100], can also be violated when the effect of base composition bias and convergence is strong. Here we found that not only can base composition bias and convergence mislead malaria parasite phylogeny using traditional concatenation methods, but also base composition convergence can mislead species tree methods by biasing the gene trees used under the multispecies coalescent model.

Both cases appear to be caused by ‘base composition attraction' between the macaque *Plasmodium* clade and the piroplasmid outgroup *T. annulata*, and this effect could be expected to occur wherever distantly related clades have converged on similar base compositions that contrast with the background base composition of the group under study. It has been suggested that the problem of non-stationarity can be addressed by simply removing the offending sites from an alignment [101], though it is important to note that in many systems, such as the malaria parasites, non-stationarity is pervasive across virtually all genes and sites. Future phylogenomic studies of clades that are characterized by strong base composition heterogeneity will benefit by testing and correcting for the effect of base composition attraction acting at all scales from supermatrices to individual gene trees, and correcting for this heterogeneity across alignments.

### 4.4. Moving forward

Using a large protein-coding sequence dataset that includes the broadest genus-level sampling of any malaria phylogeny to date, we present a novel view of the evolutionary history of the Haemosporida. We provide an empirical example of the misleading effect of base composition convergence on phylogenetic methods, and demonstrate that correction for this effect results in a topology that recovers the genus *Plasmodium* as polyphyletic with evidence for just a single invasion of malaria parasites into mammals following a single switch from sauropsid-infecting ancestors. Our analysis demonstrates that the evolution of malaria life- and natural-history traits has been more dynamic than previously appreciated, and will probably necessitate further attention as novel malaria parasite diversity continues to be discovered.

Though the present study represents the most comprehensive phylogenetic treatment of the order Haemosporida, there are still important gaps that need to be filled before a true understanding of the evolutionary relationships of this group and their traits can be assessed. *Hepatocystis kochi*, the type species of the genus/subgenus and a parasite that infects primates, not bats, should be a high priority for inclusion in future analyses. The genus *Akiba*, sometimes referred to as a subgenus of *Leucocytozoon*, but probably distinct as it uses midges and not simuliids as its vector [102], should also be a taxon of focus. We also did not include samples from several poorly known genera of lizard malaria parasites, such as *Saurocytozoon*, *Garnia* and *Fallisia*. Certainly, the marriage of morphologically identified samples of parasites with new molecular data should be continued in every case, so as to allow for taxonomic revisions that correspond to previously described species. As several genomic and transcriptomic datasets from avian haemosporidians have recently been published [40,103,104], we anticipate that these resources will be of high utility for future studies of haemosporidian phylogeny using genome-wide data.

## Supplementary Material

Supplementary Tables and Figures

## Data accessibility

All sequence data generated by this study have been deposited in GenBank (accession numbers: MF508246-MF508317, MF508319-MF508519, MF775737-MF775893). The electronic supplementary material accompanying this article contains details of samples used in this study, models of sequence evolution used in phylogenetic analyses, and results of phylogenetic analyses. Sequence alignments and phylogenetic trees have been deposited in the Dryad repository (http://dx.doi.org/10.5061/dryad.kk628dt) [105].
