# Supplementary material for: The polyphyly of Plasmodium: comprehensive phylogenetic analyses of the malaria parasites (order Haemosporida) reveal widespread taxonomic conflict
Source: R Soc Open Sci. 2018 May 23;5(5):171780. doi: 10.1098/rsos.171780 (PMC5990803; doi:10.1098/rsos.171780)
Supplement: Supplementary Tables and Figures [file rsos171780supp1.docx]

**Supplementary Table 1. Species, GenBank accession number for cytochrome b or PlasmoDB isolate name, primary host group, and data source for samples used in this study.**

| **Species** | **Cytb GenBank Accession**  **Or isolate name** | **Host group** | **Source** |
| --- | --- | --- | --- |
| *Plasmodium falciparum* | 3D7 | Mammal | PlasmoDB |
| *Plasmodium reichenowi* | CDC | Mammal | PlasmoDB |
| *Plasmodium vivax* | Sal1 | Mammal | PlasmoDB |
| *Plasmodium knowlesi* | H | Mammal | PlasmoDB |
| *Plasmodium inui* | SanAntonio | Mammal | PlasmoDB |
| *Plasmodium cynomolgi* | B | Mammal | PlasmoDB |
| *Plasmodium vinckei* | vinckei | Mammal | PlasmoDB |
| *Plasmodium chabaudi* | chabaudi | Mammal | PlasmoDB |
| *Plasmodium yoelii* | yoelii7x | Mammal | PlasmoDB |
| *Plasmodium berghei* | ANKA | Mammal | PlasmoDB |
| *Plasmodium gallinaceum* | 8A | Bird | PlasmoDB |
| *Plasmodium ovale* | curtisi | Mammal | PlasmoDB |
| *Plasmodium malariae* | UG01 | Mammal | PlasmoDB |
| *Plasmodium gaboni* | G01 | Mammal | PlasmoDB |
| *Plasmodium coatneyi* | Hackeri | Mammal | PlasmoDB |
| *Plasmodium fragile* | Nilgiri | Mammal | PlasmoDB |
| *Plasmodium* *giganteum* | LN483042 | Squamate | Borner et al. (2016) |
| *Plasmodium* sp. | LN483044 | Bird | Borner et al. (2016) |
| *Polychromophilus* sp. | LN483036 | Mammal | Borner et al. (2016) |
| *Polychromophilus* sp. | LN483038 | Mammal | Borner et al. (2016) |
| *Parahaemoproteus* sp. | LN483039 | Bird | Borner et al. (2016) |
| *Parahaemoproteus* sp. | LN483040 | Bird | Borner et al. (2016) |
| *Leucocytozoon* sp. | LN483037 | Bird | Borner et al. (2016) |
| *Leucocytozoon* sp. | LN483041 | Bird | Borner et al. (2016) |
| *Plasmodium* *koreafense* | EU834705 | Squamate | This study |
| *Plasmodium* *minuoviride* | EU834703 | Squamate | This study |
| *Plasmodium lacertiliae* | EU834710 | Squamate | This study |
| *Plasmodium* sp. (undescribed) | NA | Squamate | This study |
| *Plasmodium leucocytica* | MH177858 | Squamate | This study |
| *Plasmodium azurophilum* | AY099055 | Squamate | This study |
| *Plasmodium gemini* | EU834707 | Squamate | This study |
| *Plasmodium relictum* | MH177862 | Bird | This study |
| *Plasmodium juxtanucleare* | MH177857 | Bird | This study |
| *Plasmodium chiricahuae* | AY099061 | Squamate | This study |
| *Plasmodium mexicanum* | AY099060 | Squamate | This study |
| *Plasmodium floridense* | AY099059 | Squamate | This study |
| *Plasmodium cyclopsi* | KF159674 | Mammal | This study |
| *Hepatocystis epomophori* | KF159686 | Mammal | This study |
| *Nycteria* sp. (undescribed) | MH177856 | Mammal | This study |
| *Plasmodium odocoilei* | MH177860 | Mammal | This study |
| *Haemocystidium (Simondia) peltocephali* | KF049498 | Chelonian | This study |
| *Haemocystidium* (*Simondia*) *metchnikovi* | MH177854 | Chelonian | This study |
| *Haemocystidium mesnilium* | KF049514 | Squamate | This study |
| *Plasmodium* *mackerrasae* | EU254531 | Squamate | This study |
| *Haemocystidium* *ptyodactylii* | MH177855 | Squamate | This study |
| *Haemoproteus* *catharti* | MH177853 | Bird | This study |
| *Parahaemoproteus passeris* | MH177859 | Bird | This study |
| *Parahaemoproteus coatneyi* | EU254550 | Bird | This study |
| *Parahaemoproteus vireonis* | FJ168561 | Bird | This study |
| *Parahaemoproteus vireonis* | AY099034 | Bird | This study |
| *Parahaemoproteus sanguinis* | DQ451410 | Bird | This study |
| *Parahaemoproteus* sp*.* | MH177861 | Bird | This study |
| *Parahaemoproteus ilanpapernai* | DQ451424 | Bird | This study |
| *Haemoproteus columbae* | FJ168562 | Bird | This study |
| *Leucocytozoon fringillinarum* | MH177851 | Bird | This study |
| *Leucocytozoon majoris* | MH177852 | Bird | This study |
| *Leucocytozoon dubreuili* | AY099063 | Bird | This study |
| *Leucocytozoon simondi* | AY099064 | Bird | This study |
| *Theileria annulata* | Ankara | Mammal | EuPathDB |

**Supplementary Table 2. Genes amplified in this study, their products, and associated GenBank accession numbers.**

| **Gene ID** | **Gene** | **Product** | **GenBank Accession** |
| --- | --- | --- | --- |
| PF3D7_0206200 | “Drug_t” | Metabolite/drug transporter, putative | MF508472 - MF508490 |
| PF3D7_0318200 | Rpb1 | DNA-directed RNA polymerase subunit | MF775771 - MF775795 |
| PF3D7_0308900 | Sf3b1 | Splicing factor 3B subunit 1, putative | MF508246- MF508269 |
| PF3D7_0627800 | AACS | Acetyl-CoA synthetase, putative | MF775737 - MF775751 |
| PF3D7_0619400 | CDC48 | cell division cycle protein 48 homologue, putative | MF508407 - MF508430 |
| PF3D7_0803700 | Gtub | Tubulin gamma chain | MF508270- MF508296 |
| PF3D7_0815200 | KPNB1 | importin beta, putative | MF508363 - MF508377 |
| PF3D7_0803400 | RAD54 | DNA repair and recombination protein RAD54,putative | MF775816 - MF775838 |
| PF3D7_1017000 | POLD1 | DNA polymerase | MF508319- MF508337 |
| PF3D7_1132200 | TCP1 | T-complex protein 1 subunit alpha | MF508451 - MF508471 |
| PF3D7_1121700 | Gcn20 | PfGCN20 | MF775796 - MF775808 |
| PF3D7_1110200 | Prpf6 | Pre-mRNA-processing factor 6, putative | MF775809 - MF775815 |
| PF3D7_1219600 | ATPase2 | Phospholipid-transporting ATPase | MF775752 - MF775770 |
| PF3D7_1211700 | MCM5 | DNA replication licensing factor MCM5, putative | MF508378 - MF508406 |
| PF3D7_1213800 | ProRS | Proline--tRNA ligase | MF508297- MF508310 |
| PF3D7_1311500 | RPN7 | 26S protease regulatory subunit 7, putative | MF508311- MF508317 |
| PF3D7_1362200 | Ruvb3 | RuvB-like helicase | MF775867 - MF775893 |
| PF3D7_1361100 | Sec24a | Protein transport protein Sec24A | MF775839 - MF775866 |
| PF3D7_1475500 | Ccp1 | LCCL domain-containing protein | MF508431 - MF508450 |
| PF3D7_1410600 | “Eif2” | Eukaryotic translation initiation factor 2 gamma subunit, putative | MF508338- MF508362 |
| PF3D7_1437200 | RNR1 | Ribonucleoside-diphosphate reductase | MF508491 - MF508519 |

**Supplementary Table 3. PartitionFinder models for nucleotide and amino acid supermatrix analyses.**

| **Partition** | **Data** | **Model** |
| --- | --- | --- |
|  | **Nucleotide** |  |
| 1 | Gtub_pos1, PF3D7_1410600_pos1, MCM5 _pos1, RPN7_pos1, CDC48_pos1 | GTR+I+G |
| 2 | MCM5_pos2, PF3D7_1410600_pos2, RPN7_pos2, CDC48_pos2 | GTR+I+G |
| 3 | CCP1_pos3, RPN7_pos3, ProRS _pos3, Ruvb3_pos3, PF3D7_1410600_pos3, ATPase2_pos3, PF3D7_0206200_pos3, CDC48_pos3, Sec24a _pos3, TCP1_pos3 | GTR+I+G |
| 4 | AACS_pos1 | TVM+I+G |
| 5 | Rpb1_pos2, AACS_pos2 | TVM+I+G |
| 6 | AACS_pos3, Rpb1_pos3 | TVM+I+G |
| 7 | PF3D7_0206200_pos1, ATPase2_pos1, Prpf6_pos1, RAD54_ pos1 | GTR+I+G |
| 8 | ATPase2_pos2, RAD54_ pos2 | GTR+I+G |
| 9 | Sec24a _pos1, CCP1_ pos1 | TIM+G |
| 10 | CCP1_pos2, Prpf6_pos2, Sec24a _pos2, PF3D7_0206200_pos2 | GTR+G |
| 11 | TCP1_pos1, KPNB1_pos1, Sf3b1 _pos1 | GTR+G |
| 12 | Gtub_pos2, Sf3b1_pos2, KPNB1_pos2, TCP1_pos2 | GTR+I+G |
| 13 | Rpb1_pos1 | GTR+I+G |
| 14 | ProRS _pos1, GCN20_pos1, RNR1_pos1, POLD1_pos1, Ruvb3_pos1 | GTR+I+G |
| 15 | GCN20_pos2, POLD1_pos2, Ruvb3_pos2, RNR1_pos2, ProRS_pos2 | GTR+I+G |
| 16 | RNR1_pos3, POLD1_pos3, Sf3b1_pos3, Prpf6_pos3, KPNB1_pos3 | GTR+I+G |
| 17 | GCN20_pos3 | K81+I+G |
| 18 | MCM5 _pos3, Gtub _pos3 | GTR+G |
| 19 | RAD54_ pos3 | GTR+G |
|  |  |  |
|  | **Amino Acid** |  |
| 1 | RPN7 | JTT+G |
| 2 | AACS | JTT+I+G+F |
| 3 | CDC48 | LG4X |
| 4 | ATPase2, RAD54 | JTT+G+F |
| 5 | CCP1 | LG4X |
| 6 | Sf3b1, TCP1 | LG4X |
| 7 | Rpb1 | JTT+I+G+F |
| 8 | POLD1, GCN20 | JTT+G+F |
| 9 | Sec24a, Prpf6, PF3D7_0206200 | JTT+G+F |
| 10 | PF3D7_1410600 | CPREV+I+G |
| 11 | Gtub | LG4X |
| 12 | KPNB1 | JTT+G+F |
| 13 | MCM5 | JTT+I+G |
| 14 | ProRS | LG4X |
| 15 | RNR1 | LG4X |
| 16 | Ruvb3 | LG4X |

**Supplementary Table 4. Summary of major topological patterns found across the phylogenetic analyses performed in this study.** Shown for each analysis is the root taxon that was recovered (the lineage that was found to be sister to the rest of Haemosporida), whether *Plasmodium* was found to be polyphyletic, the most parsimonious reconstruction of the number of transitions to infecting mammalian hosts, whether *Haemoproteus* and *Parahaemoproteus* were recovered as sister lineages, and the position of the genus *Nycteria*. Shown in parentheses are bootstrap/posterior probabilities for relevant nodes.

| **Analysis** | **Root taxon** | ***Plasmodium* Monophyly** | **No. invasions of mammals** | ***Haemoproteus* and *Parahaemoproteus* sister?** | **Position of *Nycteria*** |
| --- | --- | --- | --- | --- | --- |
| **Codon Alignment Analyses** | |  |  |  |  |
| Codon alignment (unpartitioned RAxML) | Macaque *Plasmodium* | Polyphyletic | One | No | Sister to sauropsid *Plasmodium* (76) |
| Codon alignment (unpartitioned RAxML, macaque *Plasmodium* clade removed) | (*Haemoproteus*, (*Leucocytozoon*, *Parahaemoproteus* )) | Polyphyletic | One | No | Sister to sauropsid *Plasmodium* (74) |
| Codon alignment (partitioned RAxML) | (*Haemoproteus*, (*Leucocytozoon*, *Parahaemoproteus* )) | Polyphyletic | One | No | Sister to mammal *Plasmodium* (60) |
| Codon alignment (unpartitioned BEAST, strict clock) | (*Leucocytozoon*, (*Haemoproteus*, *Parahaemoproteus* )) | Polyphyletic | Ambiguous (one or two | Yes (0.99) | Sister to sauropsid *Plasmodium* (1.0) |
| Codon alignment (unpartitioned BEAST, relaxed clock) | (*Haemoproteus*, (*Leucocytozoon*, *Parahaemoproteus* )) | Polyphyletic | One | No | Sister to sauropsid *Plasmodium* (0.99) |
| Codon alignment (partitioned BEAST, strict clock) | *Leucocytozoon* | Polyphyletic | One | Yes (1.0) | Sister to sauropsid *Plasmodium* (1.0) |
| Codon alignment (partitioned BEAST, relaxed clock) | (*Leucocytozoon*, (*Haemoproteus*, *Parahaemoproteus* )) | Polyphyletic | One | Yes (0.97) | Sister to sauropsid + mammalian *Plasmodium* (0.99) |
| Codon alignment (partitioned BEAST, relaxed clock, no outgroup) | (*Leucocytozoon*, (*Haemoproteus*, *Parahaemoproteus* )) | Polyphyletic | One | Yes (0.84) | Sister to sauropsid + mammalian *Plasmodium* (1.0) |
| Codon alignment – 3^rd^ codon position removed (unpartitioned RAxML) | (*Leucocytozoon*, (*Haemoproteus*, *Parahaemoproteus* )) | Polyphyletic | One | Yes (65) | Sister to sauropsid + mammalian *Plasmodium* (71) |
| ASTRAL-II (codon alignments) | Macaque *Plasmodium* + *P. ovale*, *P. malariae* | Polyphyletic | One | Yes (0.3) | Sister to sauropsid *Plasmodium* (0.55) |
| **Amino Acid Analyses** | |  |  |  |  |
| Amino Acid (unpartitioned RAxML) | (*Leucocytozoon*, (*Haemoproteus*, *Parahaemoproteus* )) | Polyphyletic | One | Yes (62) | Sister to mammal *Plasmodium* (63) |
| Amino Acid (partitioned RAxML) | (*Leucocytozoon*, (*Haemoproteus*, *Parahaemoproteus* )) | Polyphyletic | One | Yes (50) | Sister to mammal *Plasmodium* (70) |
| Amino Acid (unpartitioned BEAST, strict clock) | *Leucocytozoon* | Polyphyletic | One | Yes (1.0) | Sister to sauropsid *Plasmodium* (0.60) |
| Amino Acid (unpartitioned BEAST, relaxed clock) | (*Leucocytozoon*, (*Haemoproteus*, *Parahaemoproteus* )) | Polyphyletic | One | Yes (0.97) | Sister to mammal *Plasmodium* (0.99) |
| Amino Acid (partitioned BEAST, strict clock) | *Leucocytozoon* | Polyphyletic | One | Yes (1.0) | Sister to sauropsid *Plasmodium* (0.59) |
| Amino Acid (partitioned BEAST, relaxed clock) | (*Leucocytozoon*, (*Haemoproteus*, *Parahaemoproteus* )) | Polyphyletic | One | Yes (0.99) | Sister to mammal *Plasmodium* (0.99) |
| Amino Acid (partitioned BEAST, relaxed clock, 21 taxa) | (*Leucocytozoon*, (*Haemoproteus*, *Parahaemoproteus* )) | Polyphyletic | One | Yes (0.99) | Sister to mammal *Plasmodium* (0.99) |
| Amino Acid (partitioned BEAST, relaxed clock, no outgroup) | *Leucocytozoon* | Polyphyletic | One | Yes (0.86) | Sister to mammal *Plasmodium* (0.99) |
| ASTRAL-II (amino acid) | (*Leucocytozoon, Parahaemoproteus , Haemoproteus, Haemocystidium*) | Polyphyletic | One | No | Sister to mammal *Plasmodium* (0.96) |
|  |  |  |  |  |  |

**Supplementary Figure 1. GC content across all genes and taxa included in this study.** A) Heat map of GC content across genes and taxa; B) Heat map of GC3 content across genes and taxa.

**Supplementary Figure 2. *Haemoproteus* is polyphyletic.** Shown is a RAxML cytochrome b gene tree for all major sauropsid haemosporidian lineages included in this study, in addition to sequences of *Haemoproteus* *antigonis* from Bertram et al. (2017). Species currently classified in the genus *Haemoproteus* are shown in red. All samples of *Haemoproteus* *antigonis* form a clade to the exclusion of other avian haemosporidians, while *H. catharti* is found more closely related to *Haemocystidium* and avian *Plasmodium* than to *H.* *columbae*. Bootstrap support values greater than 70 are shown.

**Supplementary Figure 3. *Plasmodium* *ocodoilei* is a member of an ungulate malaria parasite clade.** Shown is a RAxML cytochrome b gene tree that includes five species of malaria parasite that infect ungulates that were sequenced from Templeton et al. (2016) and Boundenga et al. (2016). The sample of *P. odocoilei* included in the present study is recovered as monophyletic with respect to these previously sequenced samples, suggesting that *P. odocoilei* is a member of an undescribed genus of malaria parasites that specializes on ungulate hosts. Bootstrap support values greater than 70 are shown.

**Supplementary Figure 4. ASTRAL-II species tree analyses.** A) ASTRAL-II analysis using gene trees generated from nucleotide codon alignments produce a topology with the macaque *Plasmodium* clade sister to the rest of the Haemosporida. B) ASTRAL-II analysis using gene trees generated from amino acid alignments produce a topology in which a large sauropsid-infecting clade is sister to all mammal-infecting malaria parasites plus the sauropsid *Plasmodium*. Local posterior probabilities are shown at nodes.
